# Late recurrence of large peri-stomal metastasis following abdomino-perineal resection of rectal cancer

**DOI:** 10.1186/1477-7819-6-96

**Published:** 2008-09-05

**Authors:** Chandrasekar Vijayasekar, Saleem Noormohamed, Mark James Cheetham

**Affiliations:** 1 Department of surgery, Royal Shrewsbury Hospital, Shrewsbury, SY3 8XQ, UK

## Abstract

**Background:**

Cutaneous metastasis from colorectal cancer after excision of the primary is a rare occurrence and presents as cutaneous or subcutaneous nodules or as a rash commonly on the anterior abdominal wall.

**Case presentation:**

This is a case description of the management of a large fungating peristomal cutaneous metastasis occurring 14 years after abdomino-perineal excision of the primary cancer. The gross appearance initially suggested possibility of a true metachronous cancer with peristomal spread. But histopathology of the resected specimen showed no colonic mucosal involvement suggesting a true large cutaneous peristomal metastasis which has not been reported previously. Literature review of presentation, management and prognosis of cutaneous metastasis from colorectal cancer is described

**Conclusion:**

Cutaneous metastasis following colorectal cancer resection is a well-recognised entity though rare. Any unusual skin lesions especially on the abdominal wall skin, previous incision scars or near the stoma should be biopsied early to rule out metastatic disease and systematic work-up should be carried out to rule out any metachronous tumour or metastasis elsewhere in the body.

## Background

Cutaneous metastasis arising from colorectal malignancy is a rare occurrence though well reported in literature. It is a pointer to more widespread disease and usually indicates a poor prognosis. Cutaneous metastasis can be the presenting feature before the primary is diagnosed e.g. Sister Mary Joseph's nodules. They can also occur late after the primary has been completely excised and can present either as cutaneous rash or as subcutaneous nodules in proximity to previous operative scars (abdominal or perineal) or on the abdominal wall skin. This is an unusual presentation of a large fungating peristomal metastasis without any visceral involvement following abdomino-perineal excision of a large T4 rectal cancer done 14 years earlier.

## Case presentation

A 61 year old lady was referred by the General Practitioner as an emergency with a rapidly enlarging fungating mass around her end-colostomy site. She had undergone an abdomino-perineal resection 14 years earlier for a Duke's B rectal cancer. She had initially presented 14 years ago with features of large bowel obstruction secondary to a large rectal tumour. She had a defunctioning loop colostomy constructed followed by adjuvant radiotherapy to shrink the rectal tumour. At the time of the Abdomino-perineal Resection, the tumour was adherent to the uterus and she underwent hysterectomy with bilateral salpingo-oopherectomy with partial vaginectomy to achieve full surgical clearance. Histopathology of the original specimen revealed a T4 tumour with clear margins and no lymph node involvement. No chemotherapy was given after surgery.

**Figure 1:**
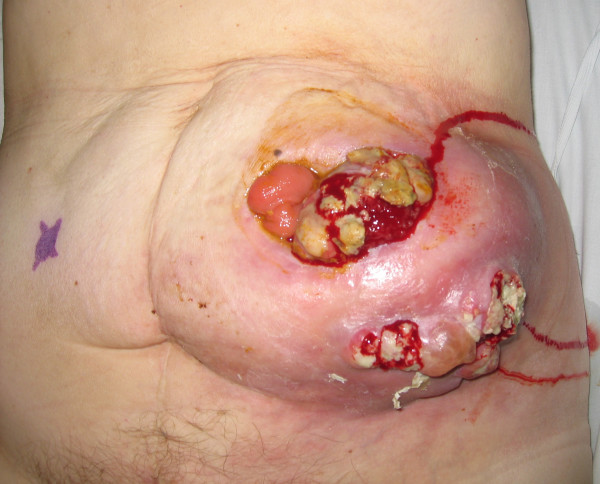
Clinical photograph showing peristomal cutaneous metastasis.

**Figure 2:**
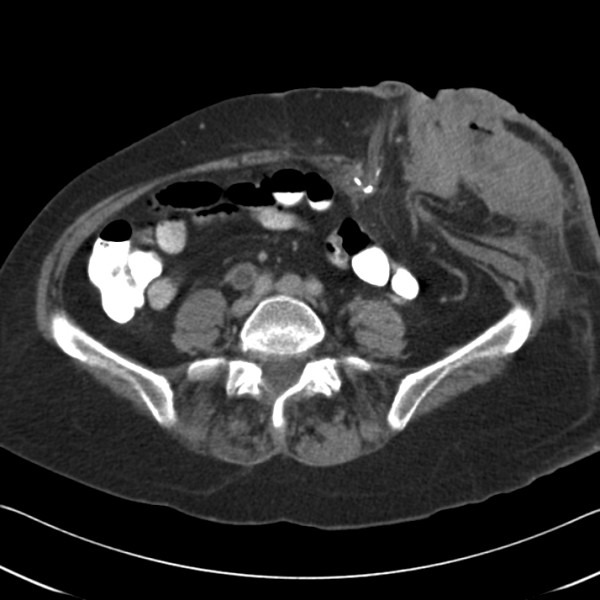
CT scan of the mass, showing abdominal wall metastasis.

She was lost to follow-up till she re-presented with this peristomal mass. On clinical examination, she was found to have a large 12 × 12 cms mass, eroding through the skin in two areas, around the stoma, which appeared stenosed but was functioning normally (Figure 1). There was no history of loss of weight or appetite and the mass had reportedly grown rapidly over a period of four weeks. Trucut biopsy of the mass gave a diagnosis of adenocarcinoma of colonic origin. Her serum CEA level was elevated at 11.1 μg/L and her Haemoglobin was 9.9 g/dL (MCV – 82.8 fL). A staging CT scan of the chest, abdomen and pelvis demonstrated the 8 × 5 cms mass adjacent to the stoma lying mainly in the subcutaneous fat with no invasion into the muscles (Figure 2). There was no evidence of any metastatic disease elsewhere.

**Figure 3:**
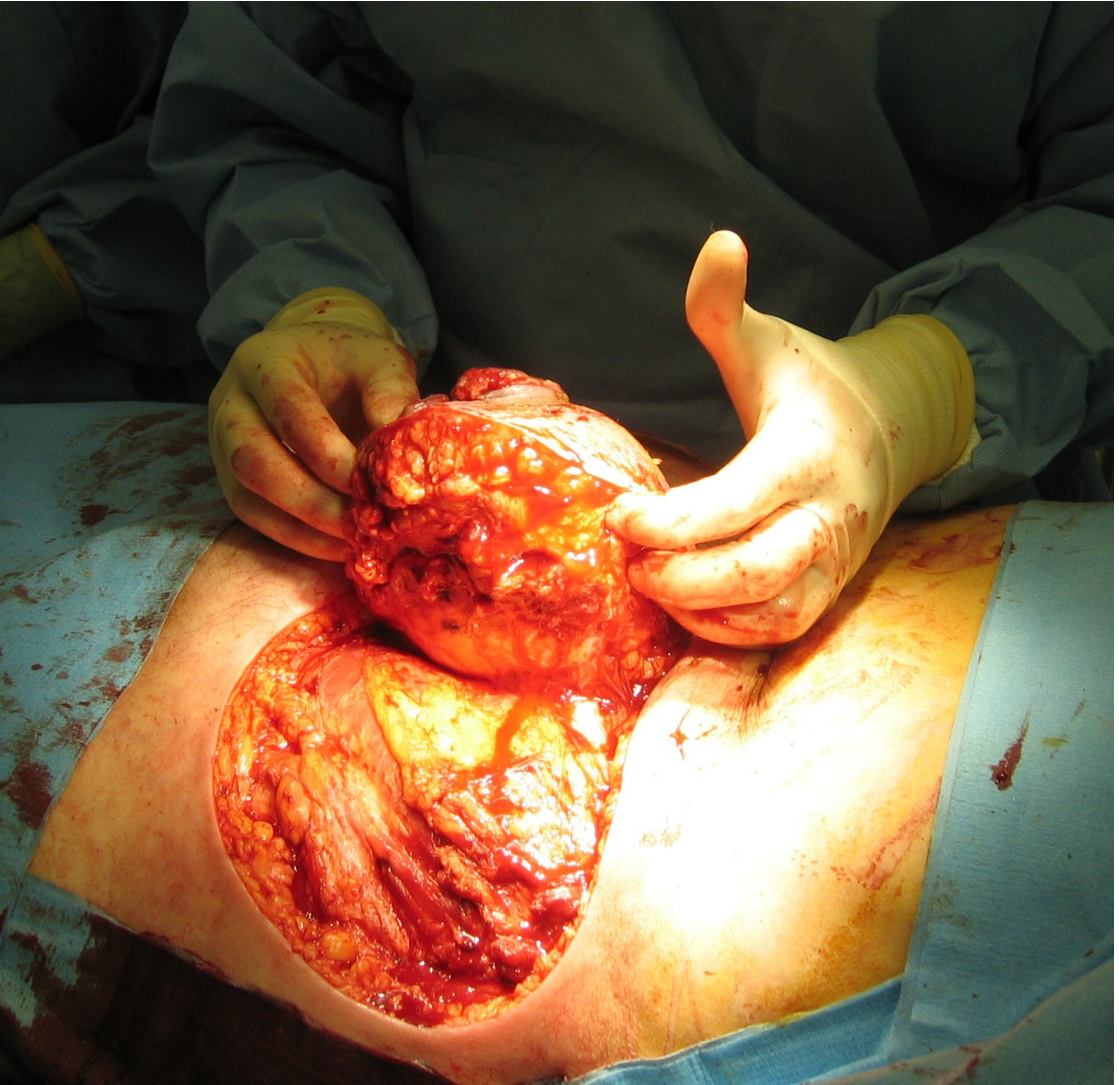
Wide local excision of the mass with the stoma.

**Figure 4:**
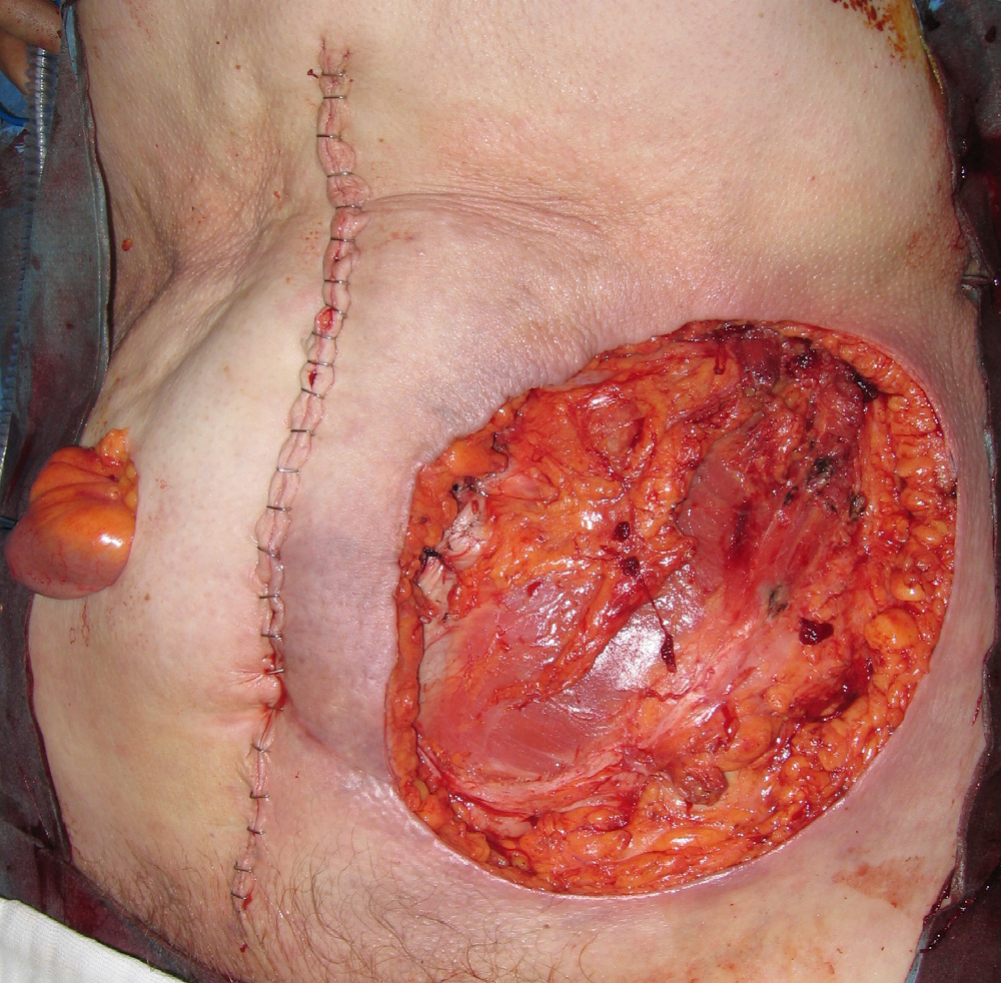
Completed excision wound with end ileostomy.

**Figure 5:**
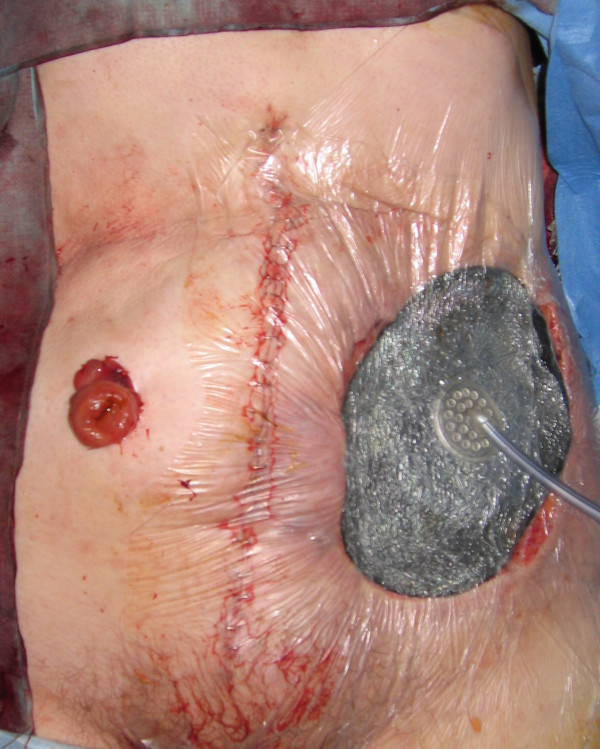
V.A.C^® ^dressing on the open wound.

At operation, the mass and the adjacent colostomy were excised wide with generous margins and placed in a bowel bag to avoid any tumour seeding (figure 3). A midline Laparotomy was then performed. There was no evidence of any intra-abdominal metastasis and a curative resection of the mass with *en bloc *completion colectomy was performed (Figure 4). The large 17 × 14 cms defect was covered with V.A.C^® ^(KCI) dressing and an end ileostomy was constructed in the right iliac fossa (figure 5). She made an uneventful post-operative recovery and after 19 days of V.A.C^® ^therapy a meshed split skin graft was harvested from the anterior thigh and used to resurface the abdominal wound.

**Figure 6:**
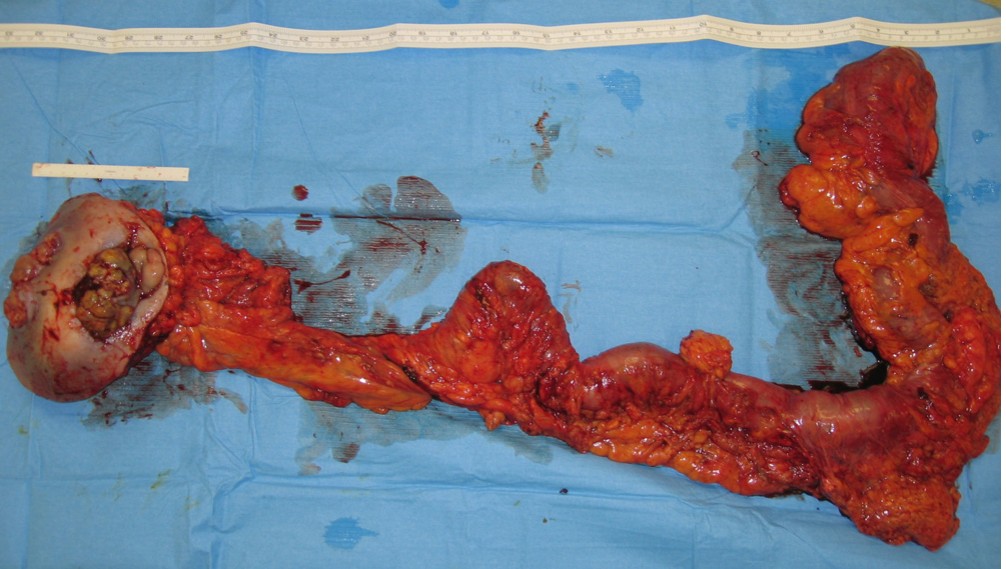
Specimen after resection.

The histopathology of the specimen showed complete excision of the subcutaneous mass with clear margins (figure 6) and the microscopic examination showed extensively necrotic and inflamed, well-differentiated colonic adenocarcinoma invading into the subcutaneous fat. There was no mucosal abnormality in the resected colon and there was no evidence of lymphovascular invasion. She was offered adjuvant chemotherapy but she declined it.

## Discussion

Stomal recurrence following abdomino-perineal resection of a rectal cancer would indicate a metachronous tumour arising from the colonic mucosa. This is well reported in world literature with at least 10 cases described [1-3]. This may present as a stomal stricture, peristomal rash or ulceration, or as a peristomal mass. The time of presentation of the metachronous tumour can be 4 to 30 years following the original resection of the primary [1]. In the present case, initially the clinical and CT appearances seemed to fit in with a metachronous tumour, explaining the reason for doing a completion colectomy and wide local excision of the mass *en bloc*. However, the histopathology of the specimen clearly shows normal colonic mucosa from caecum to the stoma with only some ulceration at the stoma site, precluding a metachronous tumour. This suggests a true isolated metastasis to the peristomal skin, as the pre-operative staging CT scan and the operative findings did not show any evidence of other visceral metastasis.

The incidence of cutaneous metastases associated with all cancers in both sexes is 0.7 to 9.0% [4]. In Lookingbill's study of metastatic disease, the incidence of cutaneous metastasis in colorectal cancer was 4.4% [5]. Colorectal metastases usually occur within 2 years of the primary tumour resection and the common organs involved are liver, peritoneum, pelvis, lung and bone in decreasing frequency [6]. The clinicopathological risk factors for skin metastasis are primary tumours that extend transmurally through the wall of the colon or rectum, lymph node metastases at presentation and perforated primary tumour [7]. The commonest site of cutaneous metastasis in colorectal cancer is the abdominal wall skin [6]. They can arise in previous surgical scars (abdominal or perineal) including colostomy reversal scar [2]. Rarely this may occur in the skin of the lower limbs, face or back [8,9]. The gross appearance of skin metastasis is usually of small subcutaneous nodules, which are under 5 cms in size, or as superficial cutaneous papules. It can present rarely as an inflammatory rash which can be confused with primary skin conditions [10,11].

Rarely colorectal metastasis may present as a large cutaneous or subcutaneous mass (> 5 cms). Alexandrescu *et al *described 2 cases of large cutaneous and subcutaneous metastases (11 cms and 5 cms) presenting in the abdominal incision scars occurring 5 and 3 years respectively after the primary tumour resection [12]. At the time of reporting, the patient had survived for 22 and 12 months respectively after wide local excision, without any other metastatic involvement. Tan *et al. *described inflammatory subcutaneous mass > 10 cms diameter appearing 22 months after primary resection, growing in size rapidly on the scapular region within a documented period of 1 month duration [13]. This was successfully excised but no information is available regarding the survival period. Greenberg *et al *reported a case of peristomal erythema, ulceration and induration appearing 6 months after abdomino-perineal resection which on shave biopsy turned out to be metastatic disease and a tiny primary focus was found in the resected stump suggesting a true synchronous tumour (< 1 year of primary resection) with peristomal cutaneous metastasis [14].

Our case is unique due to the size (> 10 cms), location (proximity to colostomy) and the time interval after resection of the primary (14 years). One of the possible mechanisms of spread of the tumour from the rectum to the colostomy site could be via the lymphatic channels in continuity with the loop colostomy that was constructed prior to the patient undergoing the abdomino-perineal excision. The second possibility is for micro-metastasis left behind in lymph nodes along the inferior mesenteric artery pedicle at the time of the abdomino-perineal resection. No specific mention was made in the initial operative notes of the curative or palliative intent of the operation, and if a high tie was carried out (ligation the inferior mesenteric artery flush with the aorta). This could lead to lymph node metastasis developing at a later date and could break through the skin presenting as a fungating mass. Histopathology did not reveal any evidence of lymphoid tissue either in the resected mass or in the remaining mesocolon of the completion colectomy to support these possible lymphatic routes. The third possible mechanism is by iatrogenic implantation of the colorectal cancer cells at the time of initial surgery. This is a well recognised problem and can present as metastatic recurrence in the midline incisions, perineal wounds, port sites and drain sites following surgery. The incidence of wound recurrence following open resection of colorectal cancer was 0.8% to 1.5% in two large series of total of 3314 patients and 80% of these recurrences occurred within 12 months of the initial surgery [15,16]. The trauma of surgery results in an inflammatory response which has been shown to enhance the successful implantation of exfoliated tumour cells in animal models. This may be a consequence of enhanced tumor cell adhesion or transient generalised immune suppression following surgery [17]. Tumour cell implantation could be the likely cause of metastatic recurrence in our case.

The survival period following diagnosis of cutaneous colorectal metastasis is 1 to 34 months. Lookingbill *et al *found a mean survival period of 18 months in 18 patients with skin metastases from colorectal carcinoma [5]. In the series by Saeed *et al*. the survival period was 2 to 4.5 months in 5 cases of colonic cancer [18]. There are case reports of patients surviving 4 years and 10.5 years following treatment of isolated metastsasis [6]. The prognosis of cutaneous metastasis would depend on the presence of concomitant metastasis elsewhere at the time of diagnosis and also on the surgical clearance achieved if found to be true isolated metastasis. Tumour differentiation and lymphovascular invasion are also important factors in altering the prognosis.

Wide local excision of the cutaneous or subcutaneous lesion is the preferred treatment option in isolated lesions. There are no clear guidelines for the optimum chemotherapeutic regimen for a non-resectable recurrence of colorectal cancer. Focal radiotherapy has been tried for cutaneous rash with poor response [8,11]. The chemotherapy treatments described include 5-fluorouracil [19], capecitabine [11], irinotecan [6], oxaliplatin [6,11,14] and cisplatin [19]. The chemotherapy drugs have evolved from a single agent 5-Fluorouracil (5-FU) as the first line agent to the current combination of drugs. The combination of irinotecan to bolus 5-FU has increased median survival from 12 months to 14.8 months [20]. The combination of infusional 5-FU and leucovorin(LV) with oxaliplatin (FOLFOX) [21] or infusional 5-FU/LV with irinotecan (FOLFIRI) [22] has increased this survival figure to above 20 months. Treating patients sequentially with FOLFIRI followed by FOLFOX, or with FOLFOX followed by FOLFIRI, has increased the median survival times to 21.5 months and 20.6 months, respectively [23]. Our patient was offered chemotherapy but she declined it. We intend to follow her up at 6 monthly intervals with CEA level estimations and cross sectional liver imaging.

## Conclusion

Cutaneous metastasis following colorectal cancer resection is a well-recognised entity though rare. Any unusual skin lesions especially on the abdominal wall skin, previous incision scars or near the stoma should be biopsied early to rule out metastatic disease and systematic work-up should be carried out to rule out any metachronous tumour or metastasis elsewhere in the body. For isolated cutaneous or subcutaneous metastasis, wide local excision would be the preferred surgical option followed by adjuvant chemotherapy depending on the histopathological status.

## Competing interests

The authors declare that they have no competing interests.

## Authors' contributions

CV prepared the draft manuscript. SN helped in literature search and preparation of manuscript. MJC conceived the idea and edited the final version for its scientific content. All authors read and approved the final manuscript.
